# Hands-On Experience of Crowdsourcing for Flood Risks. An Android Mobile Application Tested in Frederikssund, Denmark

**DOI:** 10.3390/ijerph15091926

**Published:** 2018-09-04

**Authors:** Simone Frigerio, Luca Schenato, Giulia Bossi, Matteo Mantovani, Gianluca Marcato, Alessandro Pasuto

**Affiliations:** Italian National Research Council (CNR), Research Institute for Geo-Hydrological Protection (IRPI), Corso Stati Uniti 4, 35127 Padova, Italy; simone.frigerio@irpi.cnr.it (S.F.); luca.schenato@irpi.cnr.it (L.S.); matteo.mantovani@irpi.cnr.it (M.M.); gianluca.marcato@irpi.cnr.it (G.M.); alessandro.pasuto@irpi.cnr.it (A.P.)

**Keywords:** Android application, flood risk, civil protection, DG-ECHO, preparedness, prevention

## Abstract

There is evidence that the toll of death and destruction caused by natural hazards is rising. This is often ascribed to the impact of climate change that resulted in an increased frequency of extreme meteorological events. As a consequence, it is realistic to expect that the casualties and damages caused by floods will increase in the near future. Advanced weather forecast is a fundamental tool to predict the occurrence of floods and structural mitigation measures are crucial for flood protection. However, these strategies should be associate with tools to promote and increase natural-disaster awareness and nonstructural mitigation measures in the exposed population. To bridge this gap, we coupled innovative, ICT-based technologies with crowdsourcing. The idea is to exploit geospatial data gathered by citizens and volunteers with their own devices such as mobile phones to provide authorities with relevant information in case of flood emergencies. This paper describes the design and testing of an Android application named MAppERS (Mobile Applications for Emergency Response and Support), thought to enhance active participation and response of the population in territorial and flood-risk mitigation in Frederikssund, Denmark. The results of the piloting fully validate MAppERS as an effective tool to support the decision-making process during a crisis and to improve the awareness of the community and their disaster resilience.

## 1. Introduction

Floods affect yearly at least 20 million people worldwide and claim around 20,000 lives [1]. The damages from floods will probably increase in the coming years as revealed by recent studies, linked to the predictable effect of climate change. Flood events cause adverse impacts on citizens and the economy in Europe and globally, triggering a progressive shift of responsibilities from central authorities to local communities. This variation has led to a search for new solutions within governments, concerning the proper division of roles among the state and its citizens [2].

In recent years, crowdsourcing has become an innovative ICT-based approach to solve problems, by involving active participants to obtain needed information through distributed open calls in a network of people [3,4,5,6,7]. The method is commonly adopted for active models in which information is sought directly from affected communities, or for other models in which real-time procedures like mapping, measuring, drop-down listing, and photos are outsourced to users that are randomly distributed in the territory [8]. The need for information is not often internally retrievable but known to be externally available. Hence, the emergency has to evaluate novel methods and techniques in response to a crisis, improving capacities to overcome crises, and have the ability to respond flexibly [9]. People may gather data with their own handheld devices that are appointed with GPS tracking, emphasizing the concept of user-generated content [10], able to efficiently handle any unforeseen hitches during the emergency.

This paper illustrates the development and test of MAppERS (Mobile Application for Emergency Response and Support). MAppERS consists of an Android application and a platform for snapshot data storage that allows direct analysis of data. The aim is the application of a location-based system for on-site users offering ample storage capacity of real-time data, as a helpful, relevant tool to support rescue squads on the field during a crisis, and promote awareness of the local population towards inexpensive and distributed surveys [11]. MAppERS consists of two modules: MAppERS-V (MV) for volunteers, and MAppERS-C (MC) for citizens, who are the first actors in surveillance strategies. Through the use of the app, the exposed population is trained in flood awareness and in the right use of proper terminology. Moreover, the app could be used in time of crisis to provide communication between citizens and civil-protection agents. It allows citizens to provide the localization and status of the people affected by the flood and to upload photos or geolocalized water-level assessments. This information could be extremely helpful for emergency responders to organize and prioritize interventions.

The adopted Graphical User Interface (GUI) implements a communication scheme with simple interfaces [12], while the aspired usability integrates efficiency, effectiveness, accuracy, easiness and, error tolerance [13]. The review of other existing solutions sets the layout, navigation, accessibility, icons setup, and text guidelines [14]. According to this review, the general guidelines for the design are aimed at providing users with clear, simple, and consistent conceptual structure, maximizing the effectiveness of a minimal set of graphical items and matching the presentation to the capabilities of the user [15]. This means, for example, that graphical elements on the screen should be disposed by using a grid structure, where the screen layout is standardize across all pages and where related elements are grouped together [16]. The color of the screen should be limited to 5–7 colors, with strong contrast, and also considering color-viewing deficiencies. If colors are used to group-related elements, this must be done in a consistent manner across the pages [17]. Among the different kind of icons, arbitrary icons should be avoided since they force the user to a learning process, whereas resemblance, symbolic, exemplar, and analogy icons can be used [18]. Finally, the text should be effective, efficient, and easy to learn [19]. The latest generations of mobile phones are web-enabled to upload data and, consequently, these devices may take on the role of sensors with abundant storage and computing capacity [20], that are easily accessible and powerful due to their time- and cost-effective investigations [21,22]. MAppERS supports field surveys with the feedback obtained from pilot studies tested in Frederikssund, Denmark. Both modules promote awareness, territorial knowledge, and the practice of specialized jargon to communicate hazard-relevant information to mobile phones.

In particular, this paper focuses on the design and testing of the application with specific reference to the pilot tests carried out in Frederikssund and Halsnaes (Denmark), involving rescue teams and trained citizens.

The paper has the structure as follow: Section 2 illustrates the study area, and how kits are developed. In Section 3 we describe the methodology setup, and, in Section 4, the system design is presented with case studies. Section 5 describes the output after the piloting activities with the rescue teams, and citizens voluntarily trained for data storing. In Section 6, we illustrate the procedure for a flood event, composed of clusters of services and contents performed. In Section 7, we provide a general overview and perspective.

## 2. Study Area

**Figure 1:**
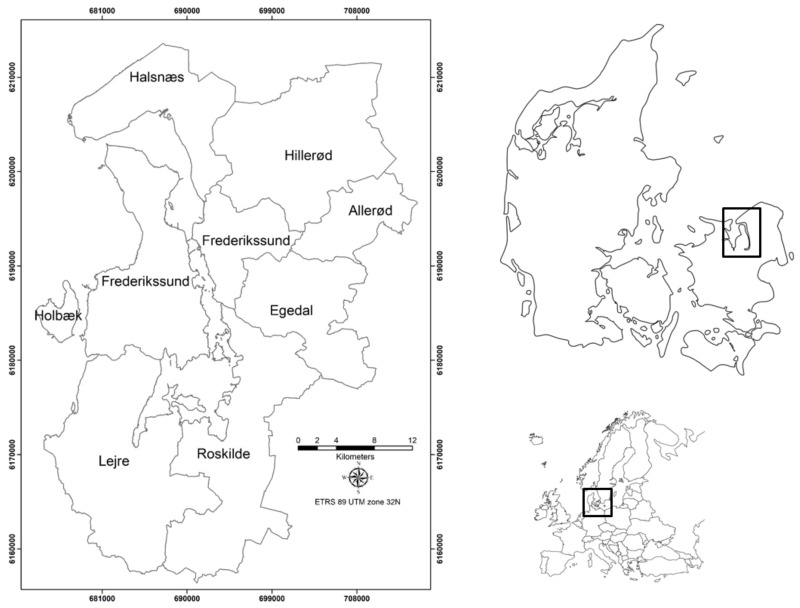
Location of the study area.

The MC and MV modules have been tested in the Frederikssund and Halsnaes municipalities on the shores of the Roskilde Fjord. They belong to the Hovedstaden Region, located in the northern part of the Zealand Island (Sjaelland) in eastern Denmark. The area covers around 382 km^2^ and 130 km of coastline with 76,000 inhabitants who were impacted by powerful storms, highlighting the need for a strict vigilance. Frederikssund and Halsnæs are part of the Frederiksborg Fire and Rescue Service (FFRS) that assists six municipalities with 250,000 of citizens and covers an area of 1058 km^2^ (Figure 1).

The organization of FFRS at a national level in Denmark depends on the Danish Emergency Management Agency (DEMA). The municipal Fire and Rescue Service must be able to provide local mitigation of accidents and disasters to prevent the harm of people, and the damage of property and the environment. In October 2013, Denmark was severely affected by two hurricanes, Allan and Bodil, causing storm surges and coastal area floods. Allan reached the Danish southern coasts in October 2013 in the form of a local storm. On that occasion, Denmark recorded its highest wind speed ever, with peak gust reaching 53.5 m/s and an average wind speed exceeding 28.5 m/s. On 5–6 December of the same year, Bodil came from the northwest with a maximum observed wind gust of 44.2 m/s. Bodil affected more significant parts of the country compared to Allan, and was even longer, with a strong W–NW wind, causing an increase of the water level by 2.06 m above the typical value. Damage to buildings, flooding, and citizen evacuation caused by hurricanes prove the need for crisis management [23]. Flooding appears as a global risk classified by the combination of likelihood and impact, and Denmark must be climate-proofed in climate-adaptation plans. As a result of the rainfall during the last few summers, the Danish Coastal Authority (Kystdirektoratet), a division of The Danish Ministry of the Environment, has proposed a systematic approach by operating national and local action plans [24]. Accordingly, municipalities must have a local action plan for climate-change adaptation within two years. The Danish Ministry of the Environment, whose role is to encourage citizens to become direct participants and municipalities to simulate natural processes, controls the plan.

## 3. Methodology

Crowdsourcing means to collect information from a “crowd” that can be either public or formed by people involved in the project. Through participation and practice, volunteers become more aware and expert about the investigation field ensuring growing quality and the credibility of the data collected [25]. Crowdsourcing offers a strategy of decentralization, as an inexpensive and quick gathering technique without being invasive to the crisis management. The crowdsourcing experience implied a low-cost solution with collecting and sharing [26]. It involved a large number of people voluntarily trained and distributed in the pilot area as a participative activity during the crisis. MAppERS methodology explores strategy, testing the frequent inability to reach populations and untapped knowledge distributed within the territory [5,27].

The goal is to catch information during a real-time flooding warning, which allows users to monitor flooding in nearby areas. MAppERS enable users to receive updates on flood alerts and provide information at specific locations by visually checking, measuring, or alerting. The application provides guidelines on every slide, relevant and upgraded by users during the first small-scale test (piloting). The paper describes an experience with an Android application composed of modules MC and MV linked to a web-based dashboard where flood-related data, events, and information can be shown and somehow forecasted.

The module (MC) integrates citizen-driven initiatives [28] through data collected for flood management. Flood mitigation includes data disseminated supporting peacetime, and when a crisis is threatening. Citizens deliver correct information voluntarily, and they can interact with measures, external instruments, reporting, location-based services, number of people exposed, geolocation of addresses, and damages of infrastructure for flood management. Before the critical phase, MC offers a toolkit to let users participate and train. To the aim of flood mitigation, people’s awareness is a long-term goal. Citizens are going to be the source of geographical information for rescue services to support the decision-making process.

By entering the MC module, the user accesses two sections: (1) a personal toolkit of preparedness and (2) real crowdsourcing activity. Their choice depends on their training and on the timeline of the crisis (vigilance or support on the field, before or during a flood event). With the personal toolkit, citizens could make their contributions visible on the dashboard. It offers location-based and continuous data from the field like the status of people, infrastructure, and measures (visible and not).

The module (MV) is bound to be used during flood emergencies by trained volunteers and has been designed and revised by civil-protection agents. The life-threat screen that allows the rescuer to signal and geolocate the presence of a life-threatening emergency is quickly available. The contribution appears immediately on the dashboard allowing the prioritization of interventions and to check the location of every volunteer. MV therefore simplifies the management of resources and roles of volunteers of Civil Protection Units (CPU), increasing the quality of on-field reports and standardizing the priority of location-based personnel (Meek et al. 2016).

The expertise of local volunteers is exploited to evaluate the impact of natural disasters [29,30]. Through the MV module, the users inspect quickly and in detail. The content is specific to events, dangers, and facilities. List can be also updated with new items, if they are missing.

The dashboard platform provides the real-time data management. It plays an essential role in data controlling and is essential during piloting to assist bug-fixing control. Users have access to a single module according to their role (MC or MV), or both (e.g., the administrator) in case of crisis managers. Once logged into the platform, users can expand or collapse groups of items on the left column where the data apply to the same criteria as in mobile screens. The tables contain the data collected by all users, which are organized with field names that match those visible in the mobile app. Users can search with various functions, selecting dates or specific criteria. A printing function is available that allows the export of a single or multiple pages providing the report function with the desired format.

## 4. Services for Flooding

The mitigation and management for floods are based on data provided by sensors that are costly and limited in their coverage [31], and a crowdsourcing-based approach is lacking on data validation. The paper introduces technology to cluster data based on temporal or spatial information, split on dissemination, and/or data analysis. The services that are described as follows implement a water-level measurement, a preparedness for flood events, a location-based upload of exposed elements, and item and message upload for the emergency.

### 4.1. Water-Level Measurement

**Figure 2:**
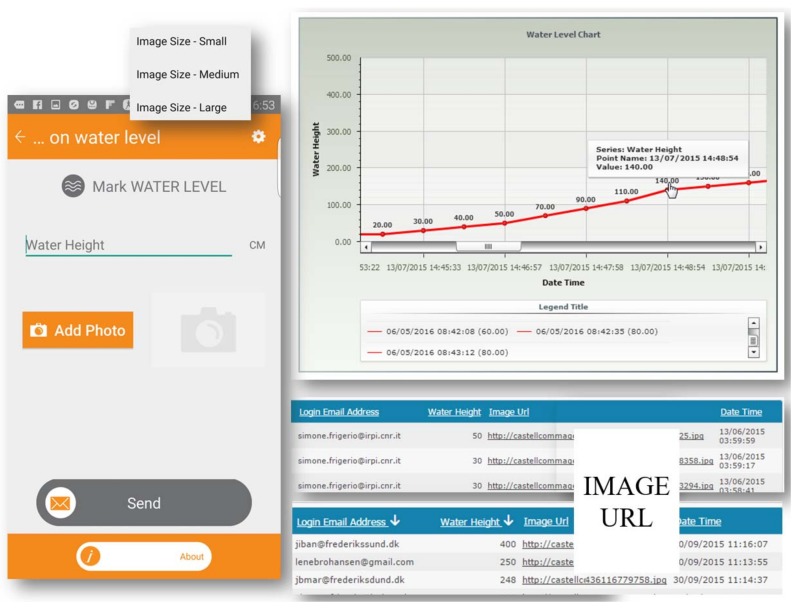
Water level marked with photo appears continuously in a chart figure.

Users profit from this tool by uploading measurements in real time, attaching a photo to validate the measurements. Choosing size from the options small, medium, or large automatically scales the file size, which is useful when bandwidth is limited. The dashboard displays measured data in real-time and makes them available for direct comparison. In Figure 2, an example of a field survey is given. The users mark the water level with height (in centimeters) and upload photos with a mobile-phone camera. The “Send” button updates the tables in MySQL DB within the dashboard by PHP Webserver. An image URL is automatically generated and it shows the collected photos. The graph in the chart continuously updates the water level and the slide bar at button allows the visualization of a specific date range. Every record collects data from all sending actions and a multiple sorting query feature allows data comparison (e.g., water height at a specific time and date validated with images).

Issues about the reliability of the water-level tool were discussed with the CPU involved in the project, from four different EU countries. All the CPU groups affirmed that an unreliable measure is better than no measure at all when working in large-scale floods. The fact that there is an integrated photo tool in the water-level section of the app is extremely valuable for rescue services, which consist mostly of local volunteers that know their territory well and could recognize places, getting a better idea of the actual flooding. Moreover, citizens were asked during training to use, if possible, measuring tapes to assess the water level.

### 4.2. Preparedness for Flood Events

**Figure 3:**
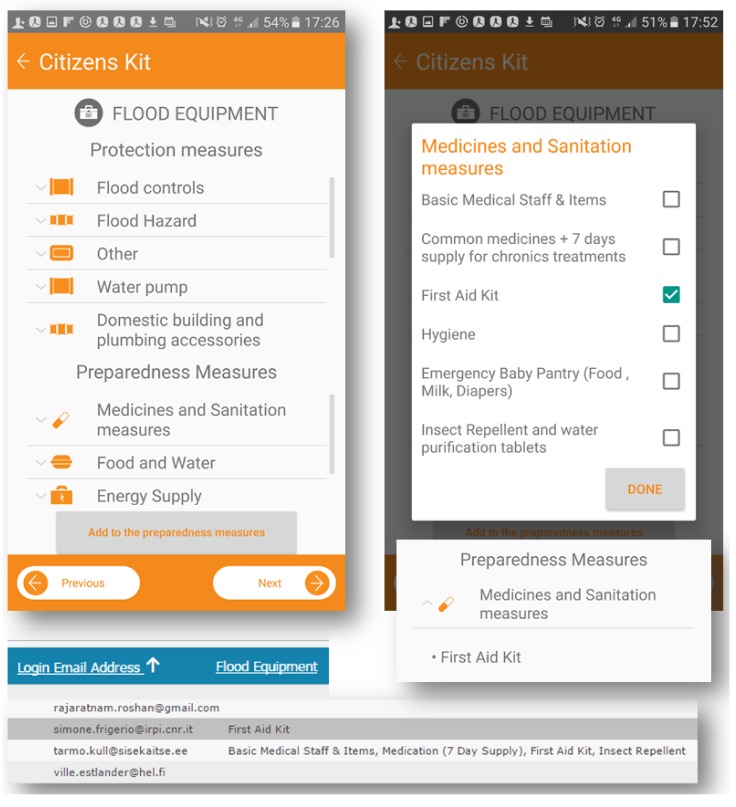
Personal kit built for flood protection and preparedness measures.

Users benefit from text and symbols ordered in drop-down lists that include standard items. The dashboard shows how single users tick the boxes, and managers of rescue services can verify individual contributions. Figure 3 shows an example of a personal kit built for flood events and based on geolocation. The methodology aims at assessing the perception of people about flood risk in detail, disseminating practices and the meaning of flood warnings.

**Figure 4:**
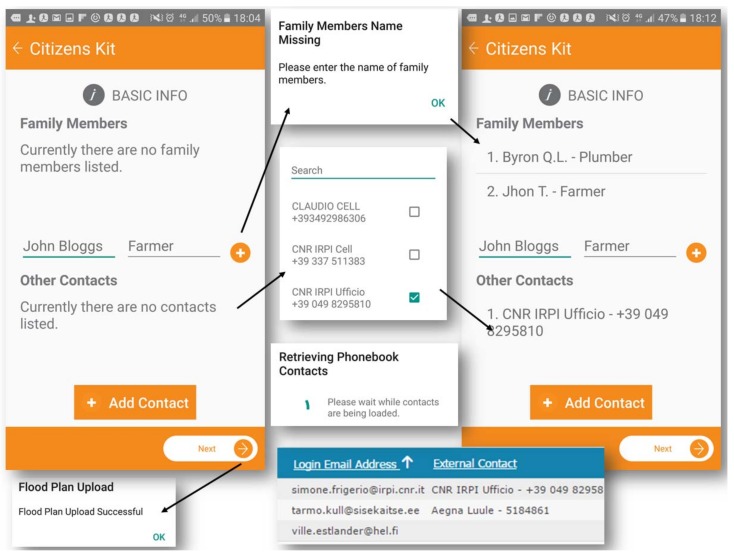
Basic info update about personal contacts.

Every user can manage the personal kit within their profile, while the comparison of users’ kits within the dashboard is a key point for the efficacy of that information. Users can visualize information on a single screen, organized in tasks of protection and preparedness; the arrows near each drop-down menu expand the list of contents, maintaining usability of the tool. Within “protection measures”, the user can find detailed guidelines, while in “preparedness measures” users can actively contribute by checking or adding new measures to their profiles (e.g., in “Medicines and Sanitation measures”, the “First Aid Kit” is the only measure marked). The kit also offers the capacity to update personal information as shown in Figure 4. Family members and other contacts can be included in the profile and added in a safety list of contacts. Retrieving phone contacts can be imported by pushing the “Add contact” button. “Previous” or “Next” offer navigation within the single screens of the citizen kit and the profile is automatically saved in the dashboard. We believe the kit is a fundamental way to promote people’s awareness and motivate them to take preventive actions before a crisis [1].

### 4.3. Location-Based Upload of Exposed Elements

**Figure 5:**
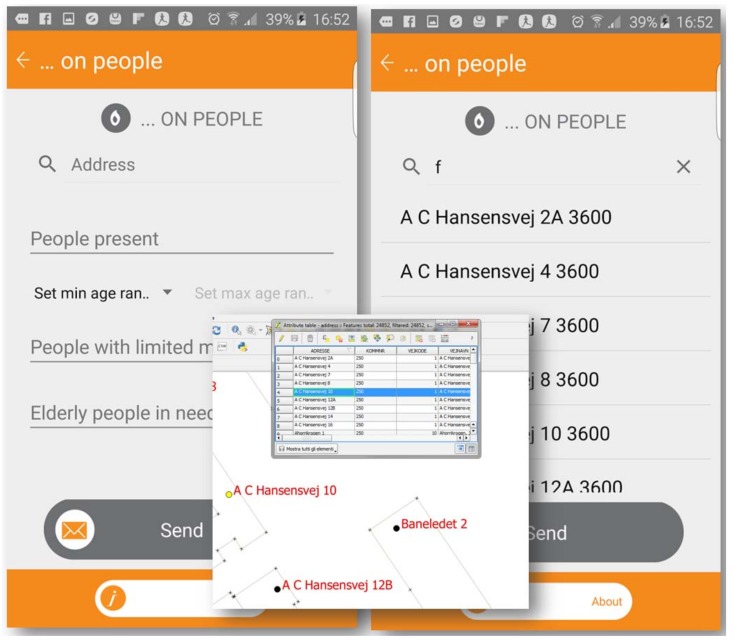
Details required for safety or specific rescue services.

The service integrates spatial data in a real-time survey to increase the involvement of the public [32]. Figure 5 shows an example of how the user can compile and upload the required information about family conditions to ensure safety in case of crisis.

**Figure 6:**
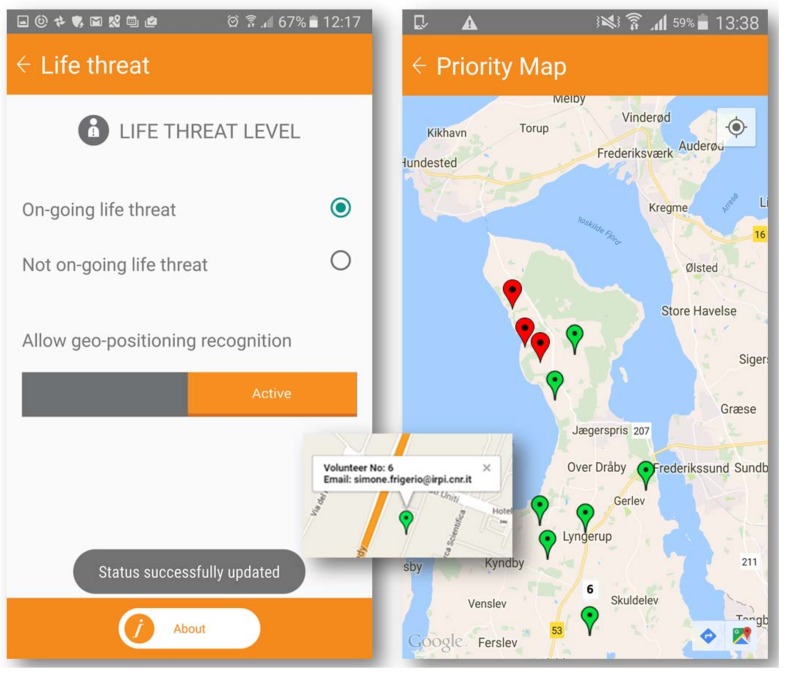
Multiusers’ geolocations with automatic clusters of visibility and ID classification.

The jQuery autocomplete service wraps the geolocated database by registry office. The data become visible in the dashboard after the “Send” button has been pressed and the crowd continuously supplies the location-based dataset (multiuser dot file), which can be exported and visualized in a GIS environment. Furthermore, the geolocations provided by GPS can be active simultaneously by multiuser access with automatically classified dots. Different clusters of visibility or classification can be customized and adapted to the aims of the kit. As an example, Figure 6 shows how geolocalized volunteers signal the presence of a life-threatening emergency in that area. Single users should maintain a proper status that is constantly updated, allowing geolocated recognition through mobile phones. The presence of a real-life threat is represented with red dots and non-on-going threats with green dots, generated by quick and easy-to-use buttons. On the screen of the priority map available to all volunteers on the field, the distribution and tracking of each phone is visible and classified by the simple criteria of life threats. Volunteers’ squads are visible with their ID code within all teams, while rescue managers can monitor all volunteers in the dashboard.

### 4.4. Item and Message Upload for Emergency

**Figure 7:**
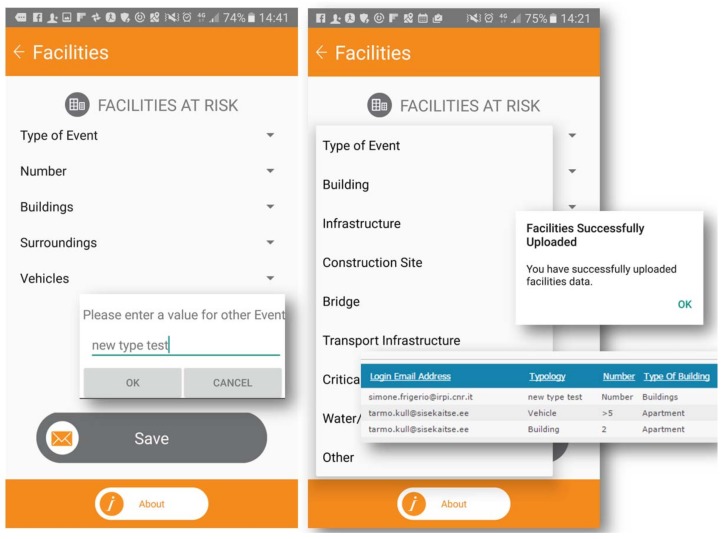
New text upload for “Type of Event” within list of facilities.

A series of drop-down items offers a detailed text and is customizable by users as the service offers a tool to upload free text. This option is available under the “Other” menu, appearing as a component in drop-down lists on several screens. A popup display appears to submit the update. Figure 7 illustrates an example in which a volunteer gathers a detailed list of facilities at risk and the environmental conditions, which could be useful for crisis support. In the list “Type of Event”, a new text enriches the menu list with confirming after “Save” button.

**Figure 8:**
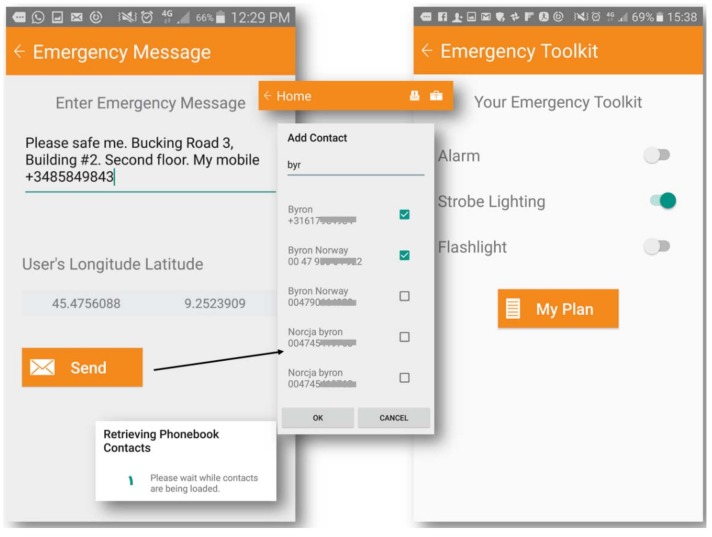
Message for emergency request, and strobe, flashlight, or alarm kit.

Furthermore, rescue managers may update the options available in the drop-down menu of the app. The option could be revised and then considered to be included as a predefined item in future versions of the application, completing the list with users’ experiences. An emergency message toolkit integrates a standard quick message, which is compiled and saved by users for an emergency request. The text can be modified within their own profile, while longitude and latitude coordinates depend on user location (Figure 8). “Retrieving phonebook contacts” retrieves the contact information from mobile phones and messages can be sent directly to multiple primary contacts selected as emergency recipients. By pressing “Send”, the real-time GPS location is included to the message. Another feature of the toolkit activates the mobile flash LED to emit a strobe pulse or a flashlight, or activates an acoustic alarm signaling an immediate emergency.

## 5. Output and Piloting

**Figure 9:**
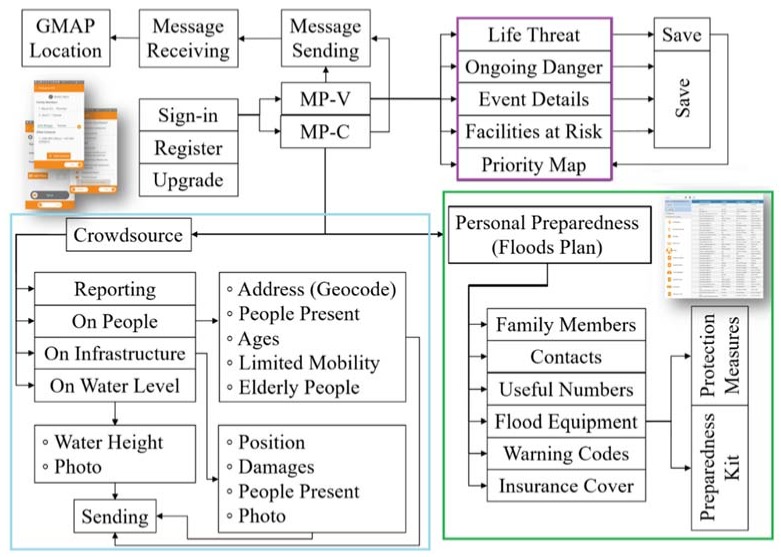
Methodology and service: in the purple cluster is the “Emergency and Rescue” section, in light blue is the “Crowdsourcing” section, and in green is the “Flood preparedness section”.

The modules MC and MV has been tested during a pilot test in which the dashboard assisted rescue services during a real-time event with the aid of volunteers combined with a promotion of long-term awareness of the recruited citizens. Citizens voluntarily joined training and piloting activities, which is fundamental to ensure proper awareness of flood events. Figure 9 illustrates the final structure built for modules, including data entry with mobile phones and data storing with the dashboard.

MC includes feedbacks of citizens as active contributions:

(1) Users provide and continuously revise location-based data of exposed people, harmonized with the local registry office and essential for rescue methods (e.g., “setting age range”, “people present”, “people with limited mobility”). Further contributions include crowd reports of damages and infrastructure, including position details (e.g., “outside the building”), typology (e.g., “electricity supplies”, “flooded basement”), and people visibly involved, linked with compulsory photos. Another screen allows marking water level, and measuring the height of water from the ground, confirming it with compulsory photos. The rescue service obtains a water-level chart, automatically updated on the dashboard, and it instantaneously compares date, time, and water height.
(2) Users are trained in personal preparedness in case of flooding as they participate as “citizen scientists” during these events. They collect data relevant for support (e.g., external contacts chosen and required to a proper phonebook), while a list of useful contacts in case of emergency is voluntarily empty during the first profile access, but organized and listed (e.g., “ambulance service”, “local fire”, “electrician”). Users can update and modify the list according to their experiences, gained during participation and involvement in past experiences [33]. These advances are visible within the dashboard. Furthermore, the screens for flood equipment present drop-down menus and cascade lists organized with home screens and widgets that are rich in content. The protection measures present short and practical lists of instruments granted by local rescue services (e.g., “flood boards”, “plastic covers”, “water pump”, “drain sealers”). Each measure has a user-friendly description for efficacy, and to illustrate their limits. Citizens profit from the mobile application by maintaining their vigilance as recruited and retained participants through their involvement during a crisis and possibly through the reputation acquired by sharing their knowledge [34]. The preparedness measures include various items that are selected by users, the choice of which uploads its plan, skipping to next screen. The module looks like a geoaware tool for lifesaving [35] to alert citizens previously trained and engaged in piloting.
(3) Users have access to a warning map screen crossing basic Google Map with their own geolocation dot. The “warning” service is active and able to import an external data source.

MV supports volunteer squads of rescue services. The application is simple and not demanding: the flowchart aims to improve and support the performance of field surveys and increase data quality. During campaigns, volunteers maintain daily routines helping in emergency events. The threat of life has variable distribution in the territory that has different priorities during decision-making. The module offers the tracking of squads showing on-going or not-present threat of life through a cascade of collected information. The update is continuous, combining life-threat signals with new real-time information. A multiuser access gathers information on the visible state of threats, geolocated associated to details of the event (e.g., type of accident, material state, oil spills, overtopping of dikes), followed by the identification of those exposed facilities (e.g., number and type of buildings, kind of surroundings, services damaged).

**Figure 10:**
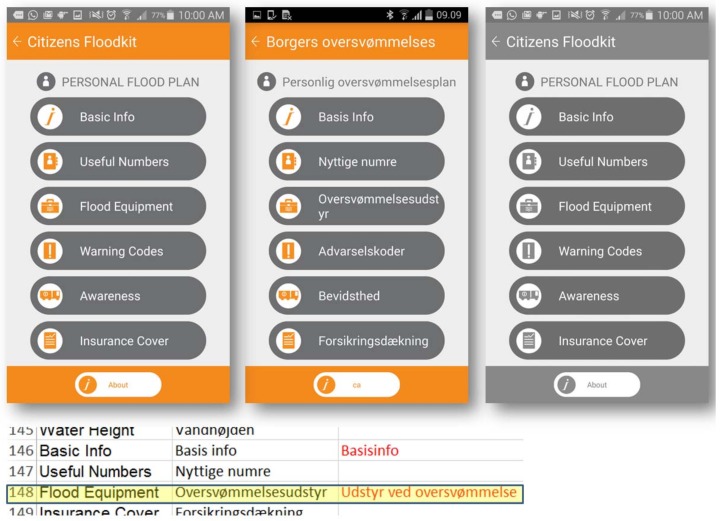
English version of screen contents and Danish translation during piloting. The third screen is the colorblind version.

MAppERS adopts the local language when installed and this setting is done automatically. The English–Danish translation for single text records was essential during the pilot phase to create a useful update of specific translations that were clear to users. During testing, the Danish text was updated for practical utility and clearness (527 records included in the text in Figure 10). The table on the bottom contains the translations for widgets on the top. The first column is the English version of the screen (e.g., “Flood Equipment”), the second column lists the Danish translation proposed by the FFRS rescue team (e.g., “Oversvømmelsesudstyr”), and the third column includes a Danish upgrade added if necessary during the pilot test (154 records updated) for increased comprehensiveness and simplicity (e.g., “Udstyr ved oversvømmelse”).

The gathered data with simulations on the field (Table 1) and MAppERS are now active for end users assembling crowd data in a continuous process through both Danish modules. During the pilot phase, MC assembled water-level and damage data from multiple measurements of the case events, completed by each user. Measurements included in Protection and Preparedness were homogeneous within all user datasets because they offered a completed long-term kit that can be updated or modified (e.g., warning codes, flood equipment, useful numbers).

The experience on the crowd is abundant on the level of damage, elements, and water field, because of data abundance, ease-of-use, and quick and rapid submission. The protection and preparedness data are less abundant because users update the database not so frequently (see Supplementary Table S1 for further details). Data records of MV are abundant since the quick field-survey options are easy and fast to use. Most of the drop-down lists profit from the final choice “other” to update consistent lists with the support of users. The idea was to hold a test to enlarge data catalogs during the pilot phase, while new options were checked by rescue teams in the field. The options that were validated were permanently included in the lists and updating is continuous. In MV the rescue squads suggested new texts for the existing lists, like type of event (e.g., collapse, accident), hazard sources (e.g., demolition), type of accident (e.g., chemical explosion, fire, leakage). New text like “other” is a checkpoint of MAppERS mobile improvement details and quality of a range of terms. Each volunteer has the responsibility to update the dataset based on events, and the rescue service has the responsibility to validate it.

## 6. System Design

The MAppERS architecture integrates an Android application, a PHP Webserver, a MySQL Database, and the Google Cloud Messaging (GCM) service. The minimum requirements are Android 4.0+, PHP 5.3+, MySQL 5.1+, internet connectivity on the mobile phone, and a web client. The Android version 7.0 was used for testing, bug-fixing, and final optimization. Android is an open-source software stack, based on the Linux kernel [37], and adopted as an operating system (OS) by a wide range of devices (86.2% of 2Q16 market share [38]). The OS encourages communities to use the open-source code and it offers the ability to customize home screens with useful widgets that enable a more natural and quicker access to functions and contents. Both iOS and Android are stable and secure leaders in mobile OS. Android appears more customizable as an open platform, of which apps can be distributed freely without extra cost.

**Figure 11:**
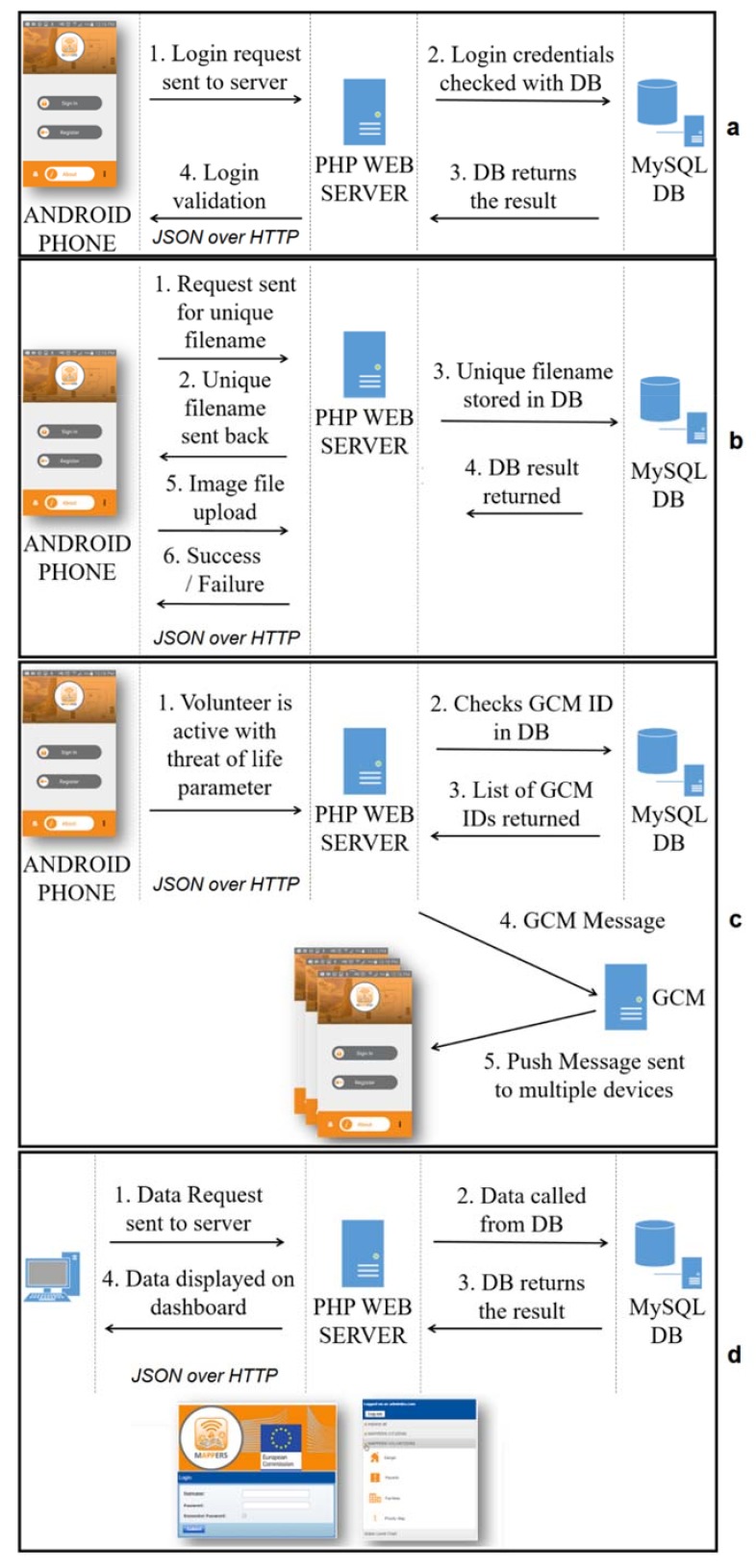
The request-response mechanism for functions of (**a**) Login/Uploading Data, (**b**) Photo Upload, (**c**) Push Notification, and (**d**) Dashboard.

The request-response mechanism draws the architecture where the Android application communicates over HTTP using JavaScript Object Notation (JSON), “an open-standard format as most common data format used for asynchronous browser/server”, to request for information with the web server, which responds over HTTP with JSON information. Cloud architecture contains a PHP Webserver, the GCM, and a MySQL DB, linking the Android application modules (MC and MV) to a dashboard for data management. The order of actions and data flow appear in four active functions, Login/Uploading Data, Photo Upload, Push Notification, and Dashboard (Figure 11).

The database structure has tables synchronized with the information collected and uploaded by specific web services listed in Table 2 (login email address is redundant and not repeated within all lists of table contents). Every web service provides uploads and responses with JSON.

## 7. Discussion

Timely and accurate information can significantly assist the emergency agency involved in flood management [39]. Flood-risk management is shifting from a primarily objective approach to an integrated methodology with attention to social aspects such as improving flood preparedness and response [40]. As a background of research on flood risk, people’s experience with flood events directly influences their behavior where they were under threat from a possible flood and in the last decades. Risk communication should be a bidirectional exchange of information based on the specific needs of people, influencing the attitudes and behavior of people within emergencies or crises and aiding decision makers. People should evaluate their risk situation, and their decisions are to be made according to preparedness and personal-safety measures. The effectiveness of communication may have a significant bearing on how people are prepared on risk. During the last decades, the risk-communication process has evolved from an engagement of learning process to a widely active role of people and the public value included in risk management.

Disaster Risk Reduction (DRR) can greatly benefit data crowsourcing in several ways: first of all, it actively contributes to overcoming the lack of information before, during, and after a disaster. Directly, the self-organized data can be digested into the DRR process. Different co-operative processes among the different actors involved in DRR can be envisaged; for example, it is also possible to obtain additional value from sharing the same data to the users with the aim of validating the data, or delivering actions or instructions among the users.

Crowdsourcing is a powerful tool for investigating distributed problems. We tested a mobile-phone application and a web platform dedicated to assisting rescue services with crisis response and subsequent recovery efforts in flooding events. MAppERS combines a setup for data control and a methodology to support crisis management. The piloting was fundamental to test the project within a study area to develop step-by-step training, test its reliability, and to upgrade the modules based on the experiences from real case studies. Both modules developed preliminary validated usefulness of the proposed approach.

The research at present is technology-driven to create an architecture with a mobile-phone application, but it is strongly devoted to the aim of deploying a crowdsourcing approach to obtain a high-quality dataset, gaining speed in acquiring information without redundancy through the productivity of trained users. The study was tested on a local scale, but is flexible and adaptable to other cases, as well as customizable to other targets due to its robust database management and multiuser control. Nowadays, several monitoring systems are implemented in hazard-prone areas [41], linking technology and local knowledge to support crisis awareness. This approach provides vast amounts of information that are only accessible by humans on the field [42]. Using the mobile application and automatic GPS tracking, users can update and correct information, enabling quick interpretation of vast amounts of data. Moreover, the possibilities of integrating the data from current legacy observational networks with data collected by crowdsourcing can pave the way for increasing the accuracy of present forecasting services and early warning systems upon proper data analysis (e.g., multivariate modeling [43]).

## 8. Conclusions

This paper shows MAppERS’ development and pilot-testing phases. The methodology that has been proposed represents an active way to support flood-risk management and rescue services. The application is neither invasive nor redundant, but it is arranged and designed considering the needs of local emergency agencies. Two modules constitute a handy gadget solution offering users a role of continuous participation to increase public awareness and personal safety. The piloting on local case studies tested MAppERS’ reliability, and crowdsourcing was expanded to allow storing of long time series to enlarge the scale and engage a higher number of communities and organizations [44]. The results of the piloting clearly show that the methodology implemented by MAppERS can effectively support the decision-making process during a crisis and improve the awareness of the community and their disaster resilience. Further effort will be paid to extend the functions of our toolkits, customizing the active services and built-in sensors to other critical cases, paying attention to the validation of the precision and accuracy of the dataset. The validation of the data may conveniently be based on one of the major cheat-detection mechanisms, such as the majority-decision or control-group approaches [45].

## Figures and Tables

**Table 1:** Data collected with first piloting experience [36].

Screen Text	Data Type	Pilot Records
MAppERS-C (MC)		
Participation	Crowdsourcing Participation	22
Exposed Elements	Crowdsourcing Exposed Elements	15
Damages	Crowdsourcing Damages	72
Water Level	Crowdsourcing Water Level	45
Basic Info	Save Family	25
Basic Info	Save External Contacts	25
Useful Numbers	Save Useful Numbers	25
Flood Equipment	Save Flood Equipment	25
Warning Codes	Save Warning Codes	21
Awareness	Save Awareness	25
Insurance Cover	Save Insurance Cover	25
MAppERS-V (MV)		
Danger	Danger	113
Hazards	Hazards	100
Facilities	Facilities	56

**Table 2:** Complete database type, organized with table, content, and web services.

Database Structure		
Table	Table Content	Web Service (with JavaScript Object Notation (JSON) Format)
Login	Login Email Address	Registration Login Forgot Password
	Password	
	User Type	
	Verified User	
Crowdsourcing Participation	Name	Crowdsourcing Participation
	Surname	
	Mobile Number	
Crowdsourcing Exposed Elements	Address	Crowdsourcing Exposed Elements
	Number of Residents	
	Age Range Min	
	Age Range Max	
	Limited Mobility	
	Elderly People	
Crowdsourcing Damages	Type of View	Crowdsourcing Damages Crowdsourcing Image Upload
	Type of Damage	
	People Involved	
	Image URL	
	Date Time	
Crowdsourcing Water Level	Water Height	Crowdsourcing Water Level
	Image URL	
	Date Time	
Save Family	Family Member	Flood Plan
Submit Family	Family Member	
Save External Contacts	External Contact	
Submit External Contacts	External Contact	
Save Useful Numbers	Useful Numbers	
Submit Useful Numbers	Useful Numbers	
Save Flood Equipment	Flood Equipment	
Submit Flood Equipment	Flood Equipment	
Save Insurance Cover	Insurance Cover	
Submit Insurance Cover	Insurance Cover	
Save Turn Off Supplier	Turn Off Supplier	
Submit Turn Off Supplier	Turn Off Supplier	
Save Warning Codes	Warning Codes	
Submit Warning Codes	Warning Codes	
User Location	Latitude	
	Longitude	
Danger	Danger Status	Danger
	Danger Visibility	
	Date Time	
Hazards	Typology	Hazards
	Hazard Sources	
	Accident Type	
	Material State	
	Material Typology	
	Spill State	
	Oil Level	
	Explosion Type	
	Date Time	
Facilities	Typology	Facilities
	Number	
	Type Of Building	
	Ground Type	
	Vehicle Type	
	Date Time	

## Supplementary Materials

The following are available online at http://www.mdpi.com/1660-4601/15/9/1926/s1, Table S1: data gathered during piloting.
